# Supplementary material for: Rabphilin-3A undergoes phase separation to regulate GluN2A mobility and surface clustering
Source: Nat Commun. 2023 Jan 24;14:379. doi: 10.1038/s41467-023-36046-6 (PMC9873702; doi:10.1038/s41467-023-36046-6)
Supplement: Supplementary file 1 — Supplementary Information [file 41467_2023_36046_MOESM1_ESM.pdf]

Supplementary information

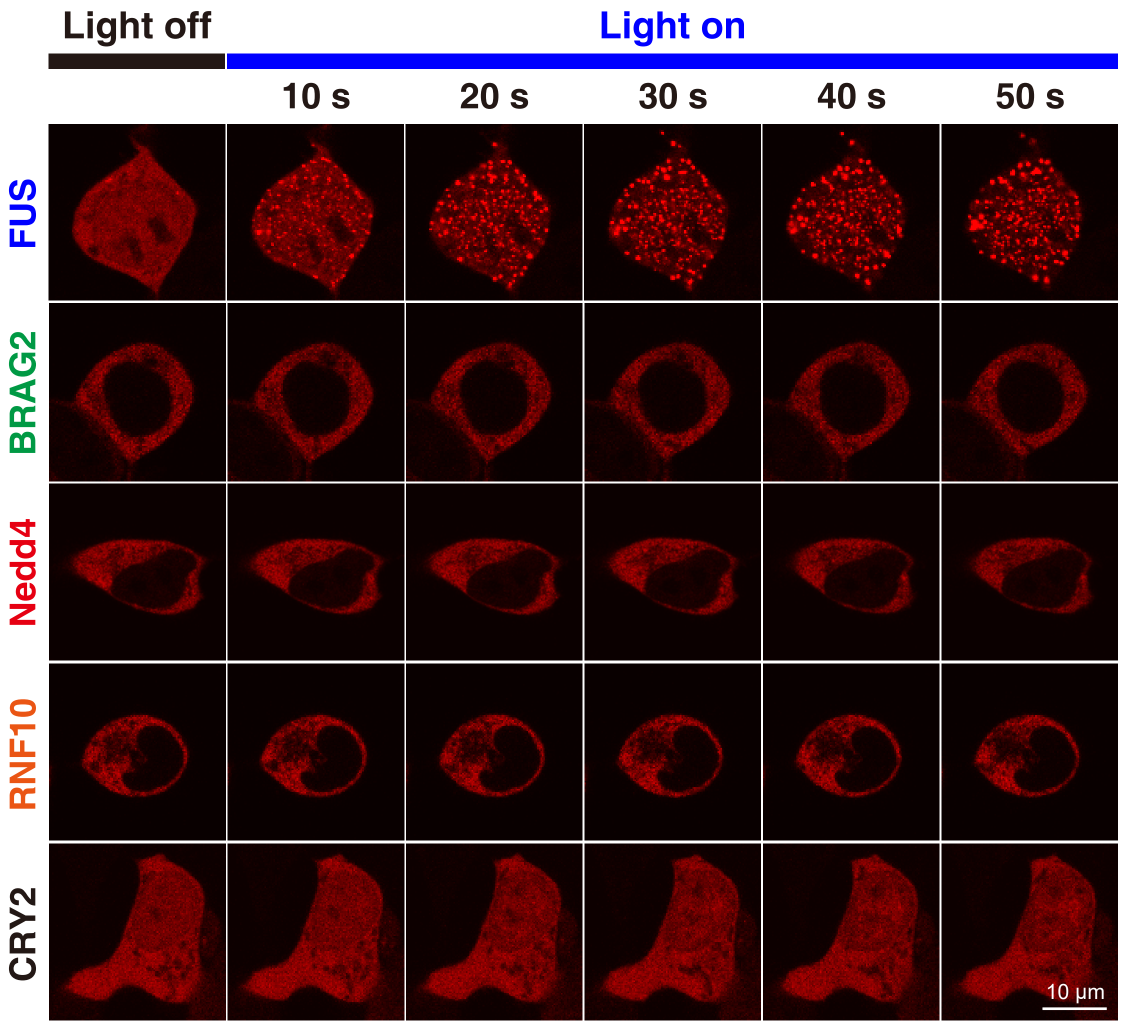

Supplementary Fig. 1: Identifying the phase separation capability of GluN2A-specific binding partners in the optoDroplet assay.

The GluN2A-specific binding partners BRAG2, Nedd4, and RNF10 were chosen for analysis in the optoDroplet assay. FUS served as a positive control, and mCherry-Cry2 alone served as a negative control.

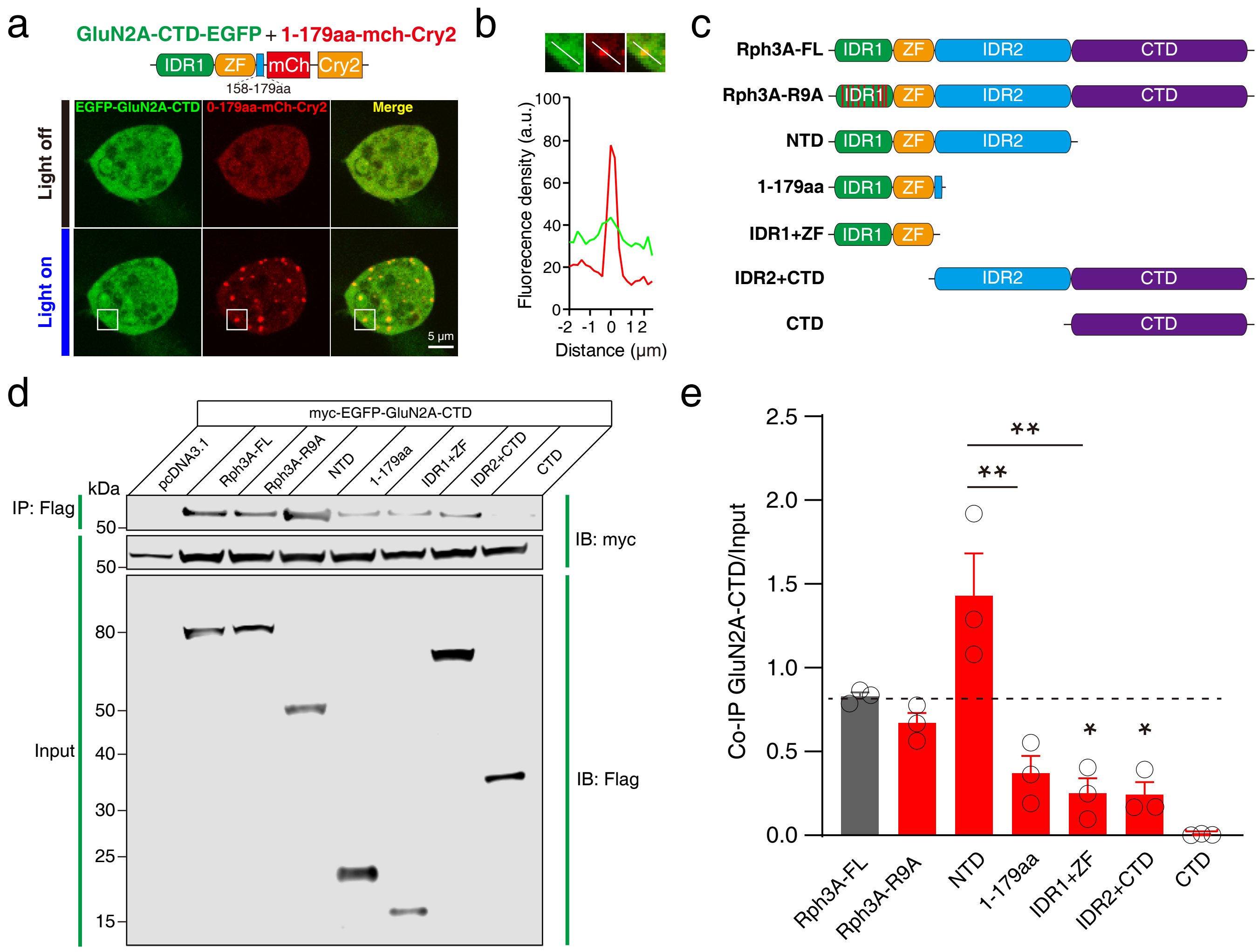

Supplementary Fig. 2: Identifying the domain of Rph3A that interacts with GluN2A CTD.

a, b Rph3A amino acids 1-179 did not condense with GluN2A-CTD in the optoDroplet assay. **c** Schematic illustration of different truncated Rph3A sequences. **d, e** Representative images and quantification of Co-IP of GluN2A-CTD with different truncated Rph3A constructs. The data are displayed as the mean ± SEM (n = 3 independent experiments for each group, **$p < 0.01$, one-way ANOVA followed by Tukey's multiple comparisons test). Source data, p values and full scan blot images of d and e are provided in the Source Data file.

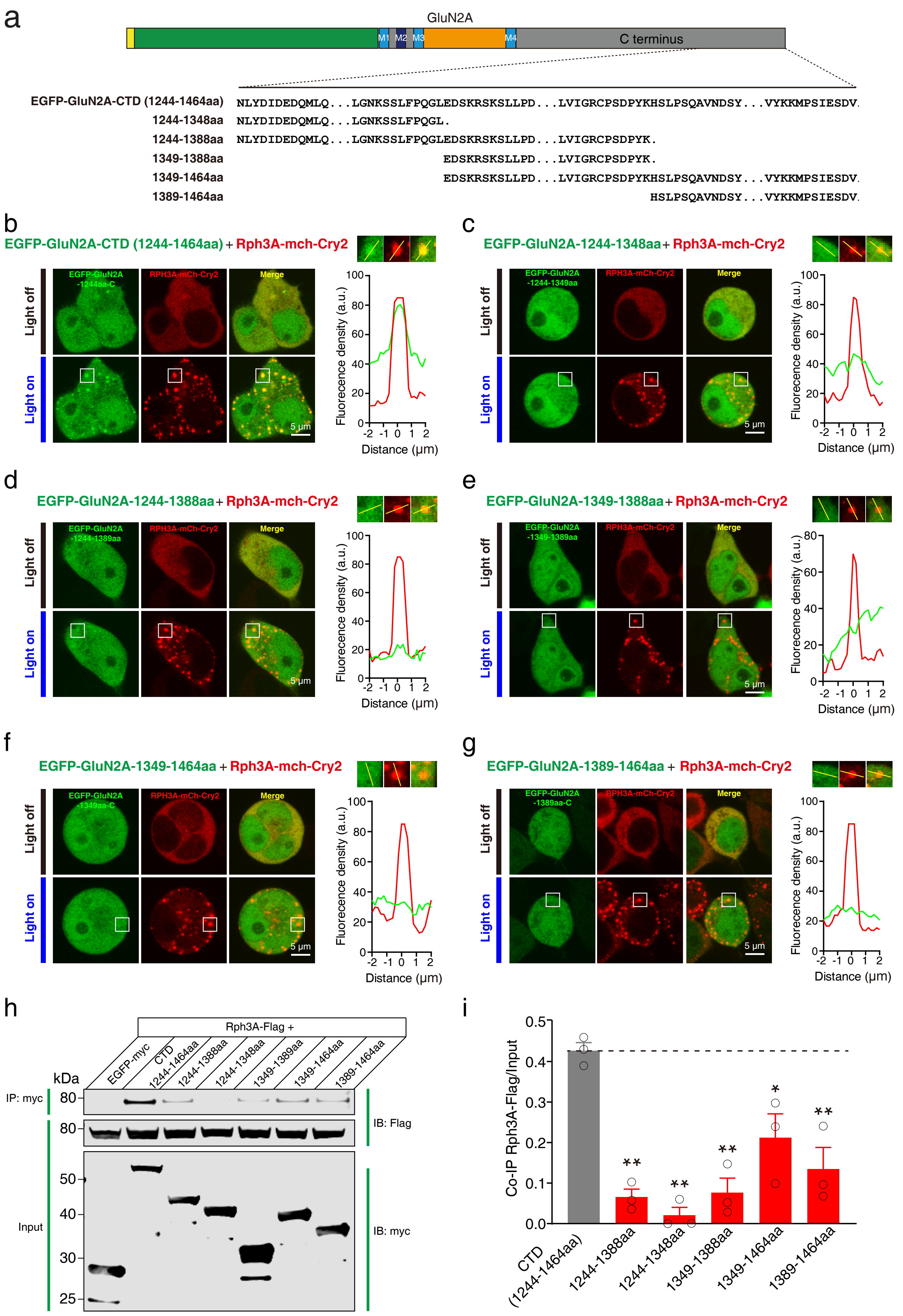
a
GluN2A
M1 M2 M3 M4
C terminus
EGFP-GluN2A-CTD (1244-1464aa) NLYDIDEDQMLQ...LGNKSSLFPQGLEDSKRSKSLLPD...LVIGRCPSDPYKHSLPSQAVNDSY...VYKKMPSIESDV.
1244-1348aa NLYDIDEDQMLQ...LGNKSSLFPQGL.
1244-1388aa NLYDIDEDQMLQ...LGNKSSLFPQGLEDSKRSKSLLPD...LVIGRCPSDPYK.
1349-1388aa EDSKRSKSLLPD...LVIGRCPSDPYK.
1349-1464aa EDSKRSKSLLPD...LVIGRCPSDPYKHSLPSQAVNDSY...VYKKMPSIESDV.
1389-1464aa HSLPSQAVNDSY...VYKKMPSIESDV.
b
EGFP-GluN2A-CTD (1244-1464aa) + Rph3A-mch-Cry2
c
EGFP-GluN2A-1244-1348aa + Rph3A-mch-Cry2
d
EGFP-GluN2A-1244-1388aa + Rph3A-mch-Cry2
e
EGFP-GluN2A-1349-1388aa + Rph3A-mch-Cry2
f
EGFP-GluN2A-1349-1464aa + Rph3A-mch-Cry2
g
EGFP-GluN2A-1389-1464aa + Rph3A-mch-Cry2
Light off
Light on
RPH3A-mCh-Cry2
Merge
5 μm
Fluorecence density (a.u.)
Distance (μm)
h
Rph3A-Flag +
EGFP-myc
CTD
1244-1464aa
1244-1388aa
1244-1348aa
1349-1389aa
1349-1464aa
1389-1464aa
kDa
IP: myc
Input
IB: Flag
IB: myc
80
50
40
30
25
i
Co-IP Rph3A-Flag/Input
CTD (1244-1464aa)
1244-1388aa
1244-1348aa
1349-1388aa
1349-1464aa
1389-1464aa
**
*

Supplementary Fig. 3: Identifying the domain of GluN2A CTD that interacts with Rph3A.

a Schematic illustration of different truncated GluN2A-CTD constructs. **b-g** Amino acids 1244-1464 of GluN2A CTD condensed with Rph3A in the optoDroplet assay, whereas none of the truncated GluN2A CTD constructs did so. **h, i** Representative images and quantification of Co-IP of Rph3A with different truncated GluN2A CTD constructs. The data are displayed as the mean ± SEM (n = 3 independent experiments for each group, $**p < 0.01$, one-way ANOVA followed by Tukey's multiple comparisons test). Source data, p values and full scan blot images of h and i are provided in the Source Data file.

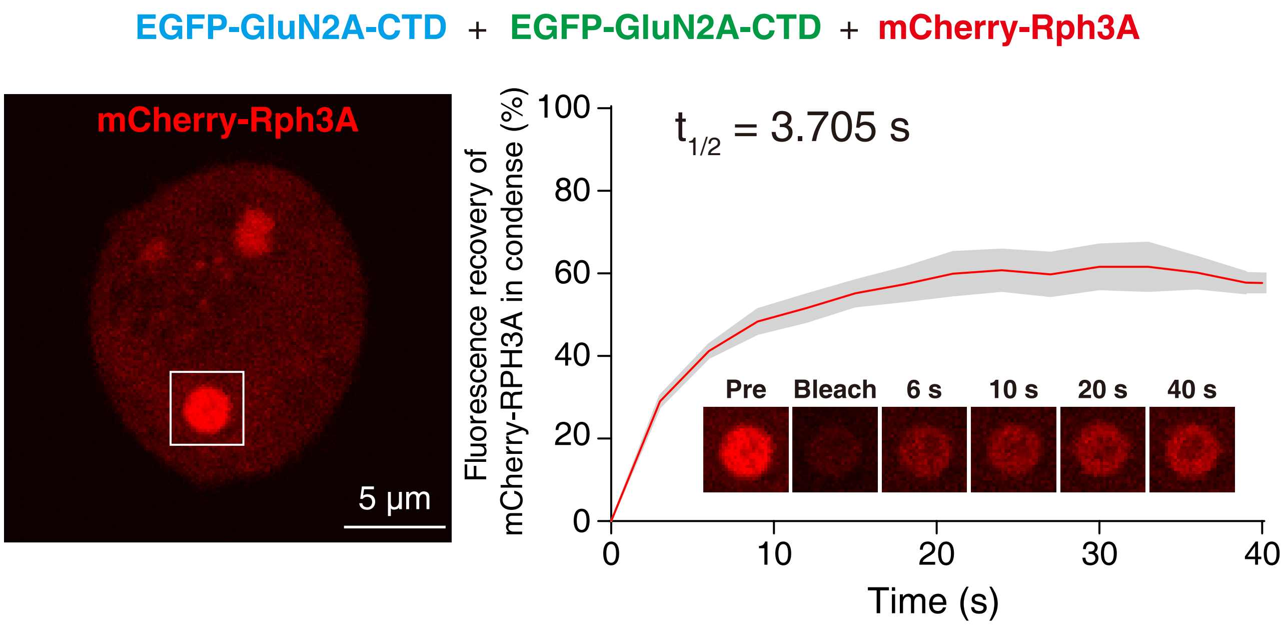

Supplementary Fig. 4: Rph3A forms liquid condensates with PSD95 and GluN2A CTD in HEK293 cells.

Representative images and quantification of fluorescence recovery from the FRAP analysis of mCherry-Rph3A condensates in HEK293 cells. The data are displayed as the mean ± SEM (n = 5 puncta). Source data are provided in the Source Data file.

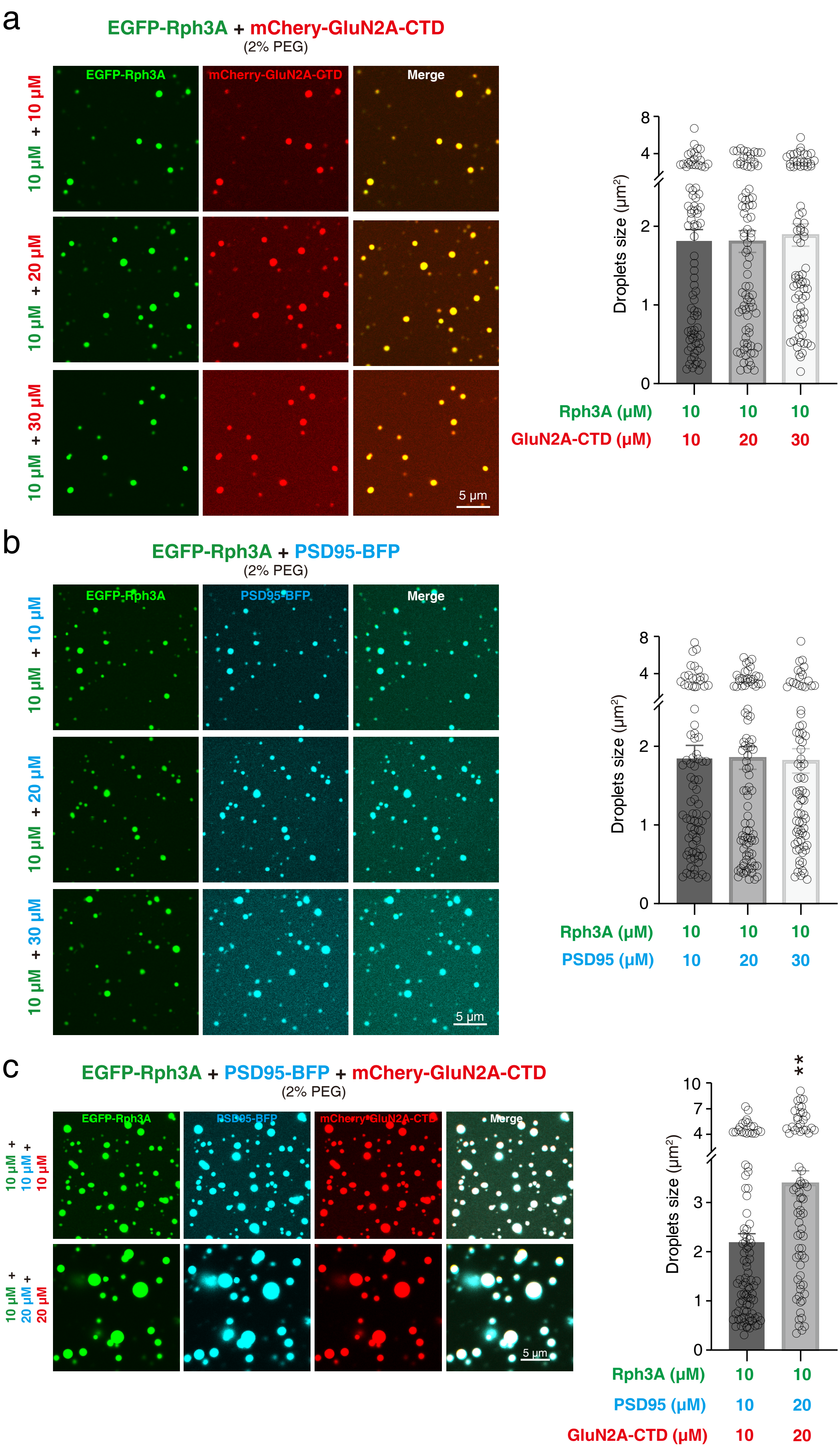
a
EGFP-Rph3A + mChery-GluN2A-CTD
(2% PEG)
EGFP-Rph3A
mCherry-GluN2A-CTD
Merge
10 μM + 10 μM
10 μM + 20 μM
10 μM + 30 μM
5 μm
Droplets size (μm²)
Rph3A (μM) 10 10 10
GluN2A-CTD (μM) 10 20 30
b
EGFP-Rph3A + PSD95-BFP
(2% PEG)
EGFP-Rph3A
PSD95-BFP
Merge
10 μM + 10 μM
10 μM + 20 μM
10 μM + 30 μM
5 μm
Droplets size (μm²)
Rph3A (μM) 10 10 10
PSD95 (μM) 10 20 30
c
EGFP-Rph3A + PSD95-BFP + mChery-GluN2A-CTD
(2% PEG)
EGFP-Rph3A
PSD95-BFP
mCherry-GluN2A-CTD
Merge
10 μM + 10 μM + 10 μM
10 μM + 20 μM + 20 μM
5 μm
**
Droplets size (μm²)
Rph3A (μM) 10 10
PSD95 (μM) 10 20
GluN2A-CTD (μM) 10 20

Supplementary Fig. 5: The GluN2A/PSD95 complex promotes the phase separation of Rph3A in a concentration-dependent manner.

a Representative images and quantification of droplets composed of Rph3A and GluN2A-CTD at different concentrations (GluN2A-CTD 10 μM: n = 83 droplets; GluN2A-CTD 20 μM n = 82 droplets; GluN2A-CTD 30 μM n = 76 droplets, $p = 0.93$, one-way ANOVA). **b** Representative images and quantification of droplets composed of Rph3A and PSD95 at different concentrations (PSD95 10 μM: n = 81 droplets; PSD95 20 μM: n = 92 droplets; PSD95 30 μM: n = 77 droplets, $p = 0.98$, one-way ANOVA). **c** Representative images and quantification of droplets composed of Rph3A, GluN2A-CTD, and PSD95 at different concentrations (GluN2A-CTD/PSD95 10 μM: n = 92 droplets; GluN2A-CTD/PSD95 20 μM: n = 72 droplets, **$p < 0.01$, two-tailed unpaired t test). Source data and p values of a, b and c are provided in the Source Data file.

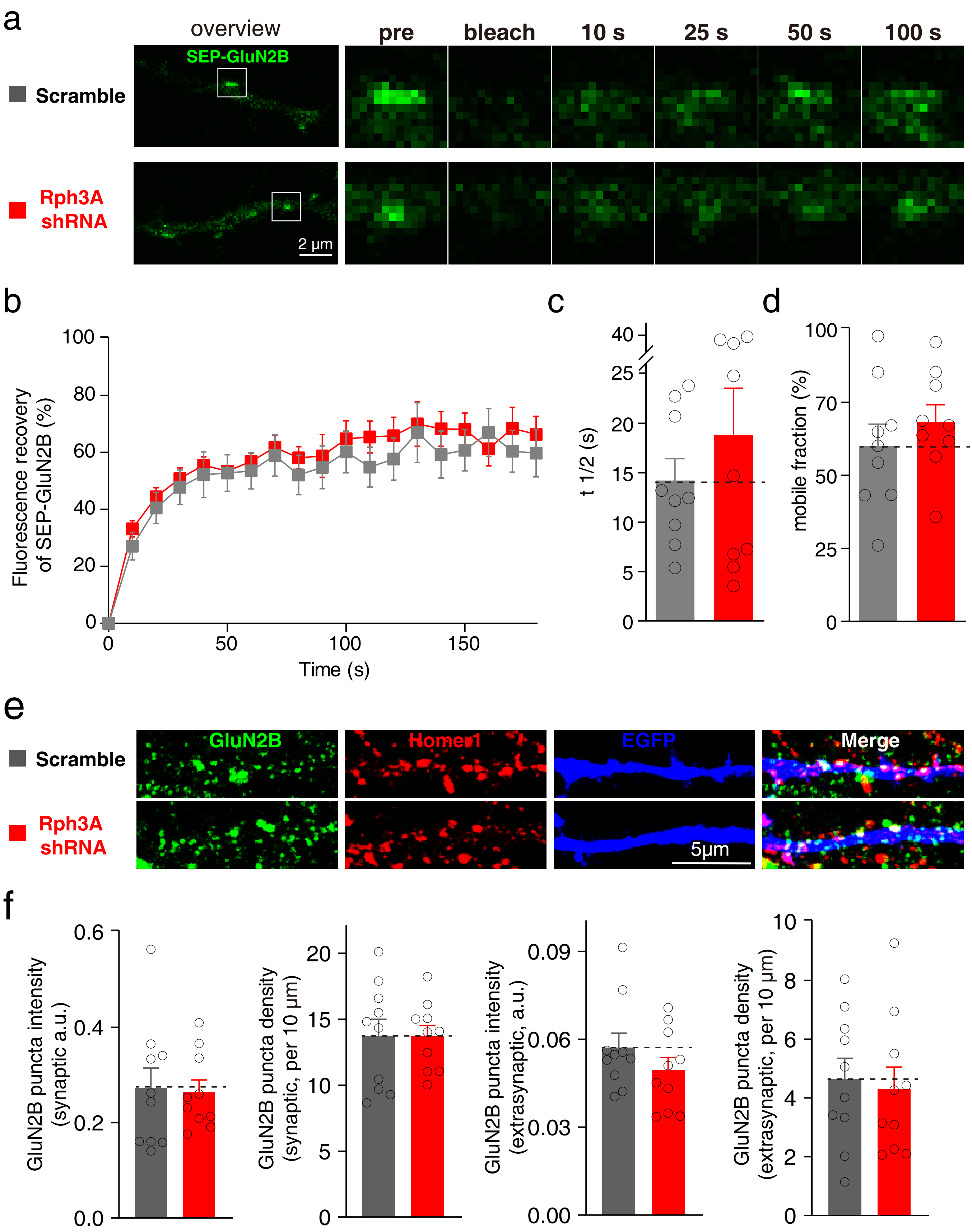

Supplementary Fig. 6: Rph3A knockdown did not affect the mobility or surface clustering of GluN2B.

a Representative FRAP images of SEP-GluN2A in Rph3A-knockdown hippocampal neurons. **b** Quantification of the fluorescence recovery shown in a. The data are

displayed as the mean ± SEM (n = 9 puncta for each group). **c** The recovery rate was calculated as the half-time of the recovery curve in b. The data are displayed as the mean ± SEM (n numbers are defined in b, $p = 0.39$, two-tailed unpaired t test). **d** The mobile fraction of GluN2A was calculated based on the plateau of the recovery curve in b. The data are displayed as the mean ± SEM (n numbers are defined in b, $p = 0.40$, two-tailed unpaired t test). **e, f** Representative images and quantification of synaptic and extrasynaptic GluN2B in Rph3A knockdown hippocampal neurons. The data are displayed as the mean ± SEM (n = 10 dendrites from 5 neurons for each group, two-tailed unpaired t test). The full images of a and e are showed in Supplementary Fig. 13. Source data and p value of b, c, d and f are provided in the Source Data file.

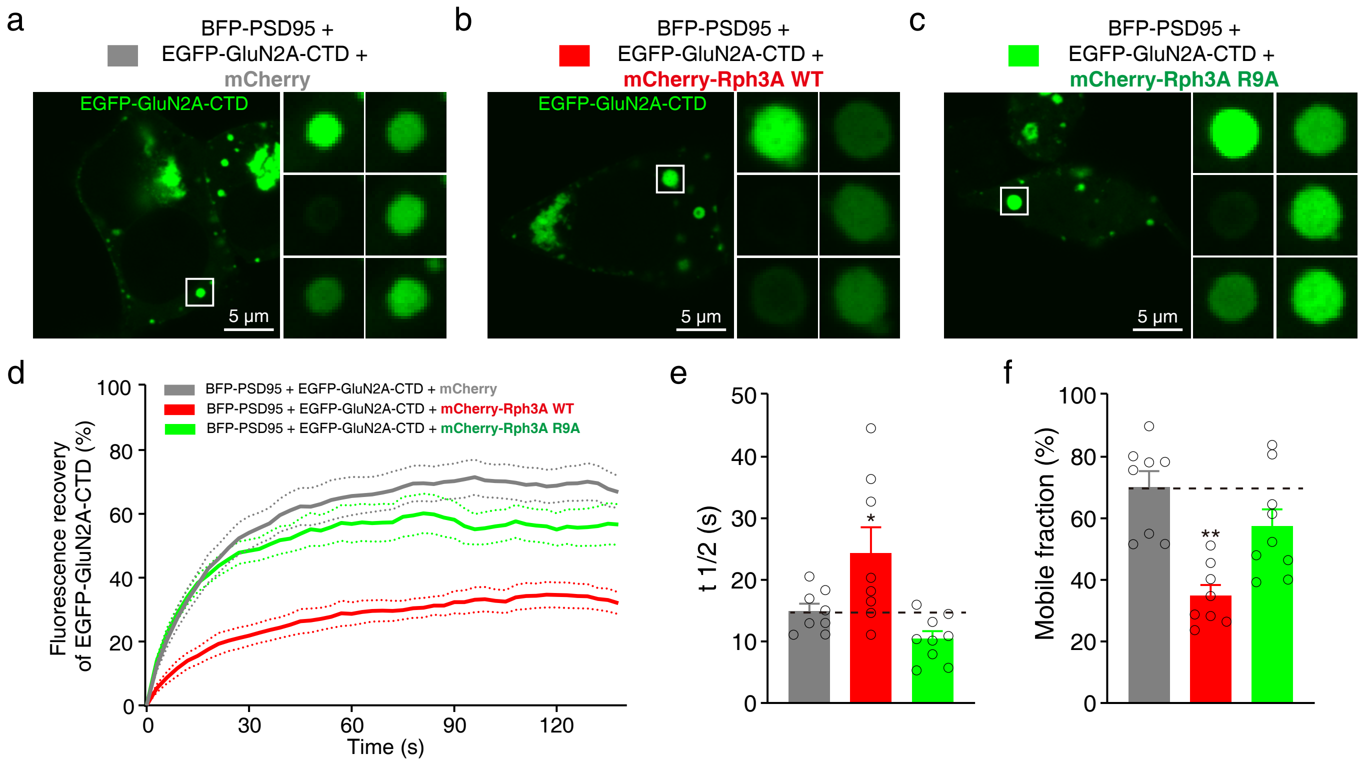

Supplementary Fig. 7: Rph3A phase separation decreased the mobility of the GluN2A CTD in HEK293 cells.

a-c Representative images from the FRAP of EGFP-GluN2A-CTD in HEK293 cells expressing fluorescent protein-tagged PSD95, GluN2A-CTD, WT, and R9A Rph3A. **d** Quantification of the fluorescence recovery shown in a-c. The data are displayed as the mean ± SEM (mCherry: n = 8 puncta; mCherry-Rph3A WT: n = 8 puncta; mCherry-Rph3A R9A: n = 9 puncta). **e** The recovery rate was calculated as the half-time of the recovery curve in d. The data are displayed as the mean ± SEM (n numbers are defined in d, *$p < 0.05$, one-way ANOVA followed by Tukey's multiple comparisons test). **f** The mobile fraction of GluN2A-CTD was calculated based on the plateau of the recovery curve in d. The data are displayed as the mean ± SEM (n numbers are defined in d, **$p < 0.01$, one-way ANOVA followed by Tukey's multiple comparisons test). Source data and p value of d, e and f are provided in the Source Data file.

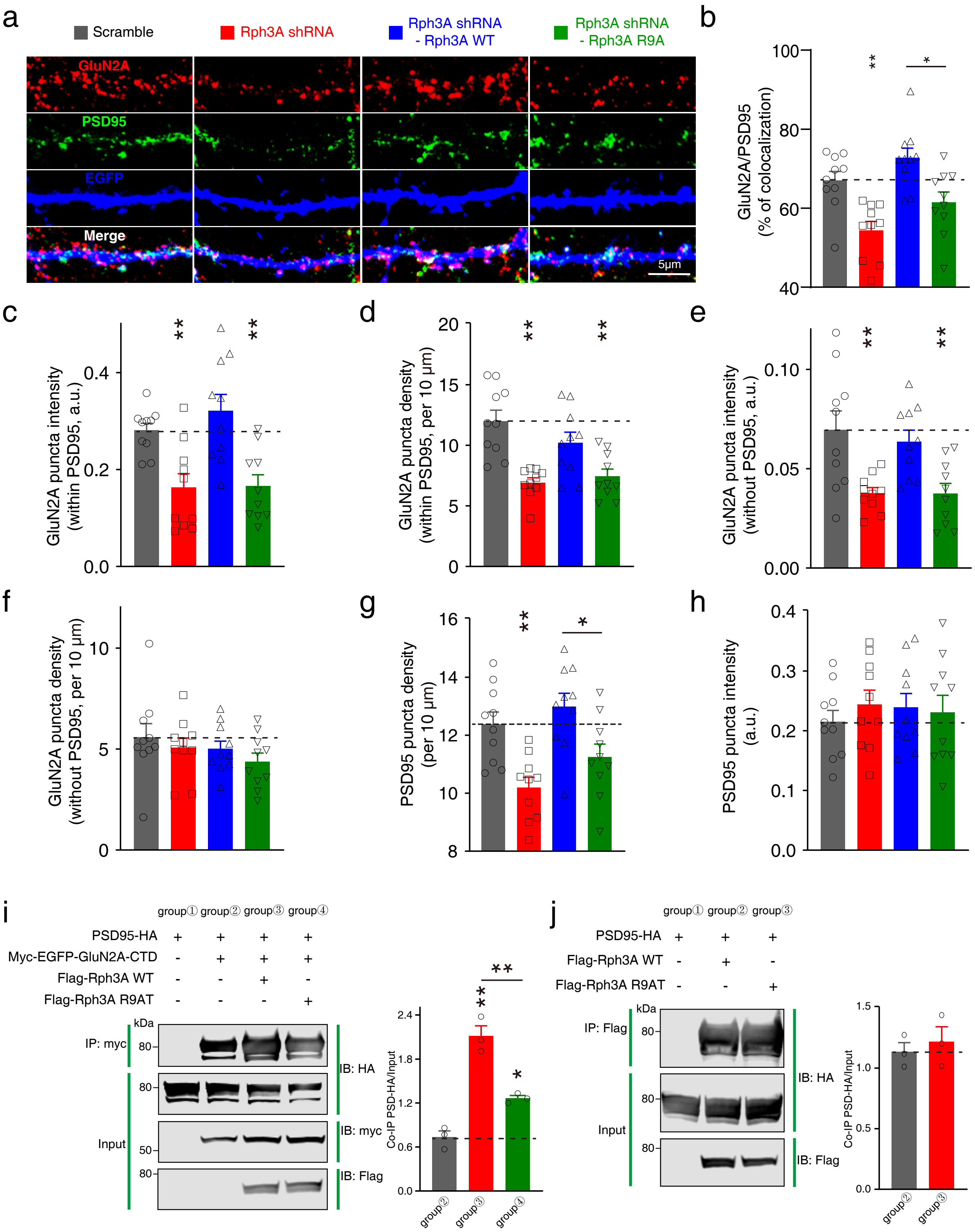

Supplementary Fig. 8: Disruption of the phase separation capacity of Rph3A impaired GluN2A/PSD95 complex formation.

a Representative images of surface GluN2A and PSD95 staining in Rph3A-knockdown and Rph3A-reexpressing neurons. **b-f** Quantification of the percentage of

GluN2A clusters colocalized with PSD95 and the fluorescence intensity and density of GluN2A clusters with or without PSD9 5 in a. The data are displayed as the mean ± SEM (n = 10 dendrites from 5 neurons for each group, *p < 0.05, **p < 0.01, one-way ANOVA followed by Tukey's multiple comparisons test (b), or by Dunnett's multiple comparisons test (c–f)). **g, h** Quantification of the fluorescence intensity and density of PSD95 clusters in a. The data are displayed as the mean ± SEM (n numbers are defined in b, *p < 0.05, **p < 0.01, one-way ANOVA followed by Tukey's multiple comparisons test (g), or by Dunnett's multiple comparisons test (h)). **i** Representative images and quantification of Co-IP of PSD95 with GluN2A -CTD in the presence of WT or R9A Rph3A. The data are displayed as the mean ± SEM (n = 3 independent experiments for each group, *p < 0.05, **p < 0.01, one-way ANOVA followed by Tukey's multiple comparisons test). **j** Representative images and quantification of the results of Co-IP of PSD95 with WT or R9ARph3A. The data are displayed as the mean ± SEM (n = 3 independent experiments for each group, p = 0.58, two-tailed unpaired t test). The full images of a are showed in Supplementary Fig. 13. Source data, p values and full scan blot image of b–j are provided in the Source Data file.

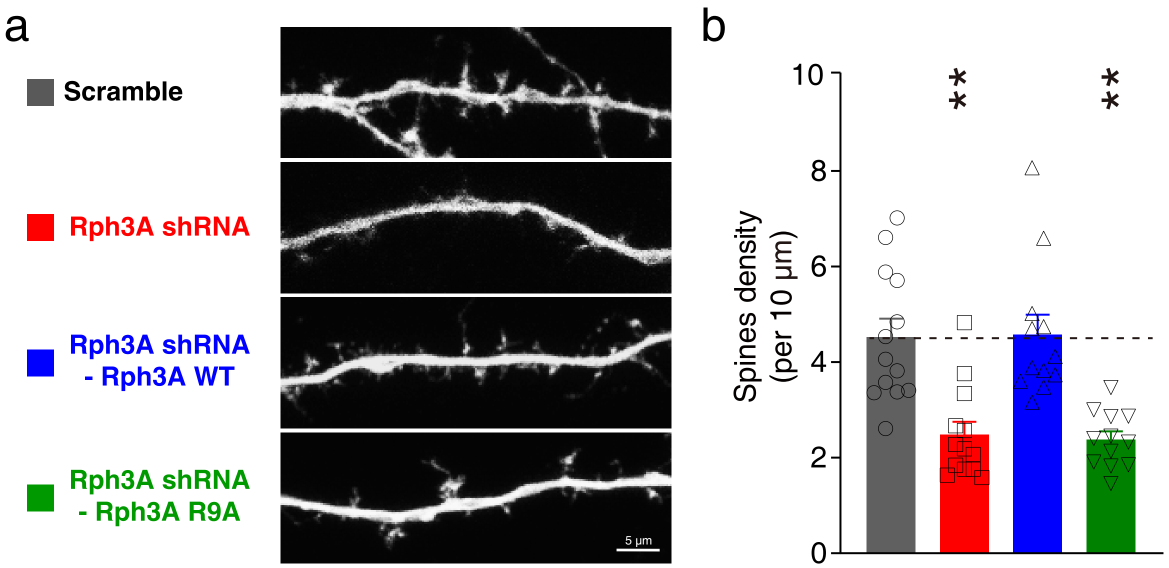

Supplementary Fig. 9: Phase separation of Rph3A was associated with a normal spine density in cultured hippocampal neurons.

a Representative images and **b** quantification of spine density in Rph3A-knockdown and Rph3A-re-expressing neurons. The data are displayed as the mean ± SEM (Scramble: n = 13 dendrites from 7 neurons; Rph3A shRNA: 13 dendrites from 7 neurons; Rph3A shRNA-Rph3A WT: 12 dendrites from 6 neurons; Rph3A shRNA-Rph3A R9A: 12 dendrites from 6 neurons, $**p < 0.01$, one-way ANOVA followed by Dunnett's multiple comparisons test). The full images of a are showed in Supplementary Fig. 13. Source data and p values of b are provided in the Source Data file.

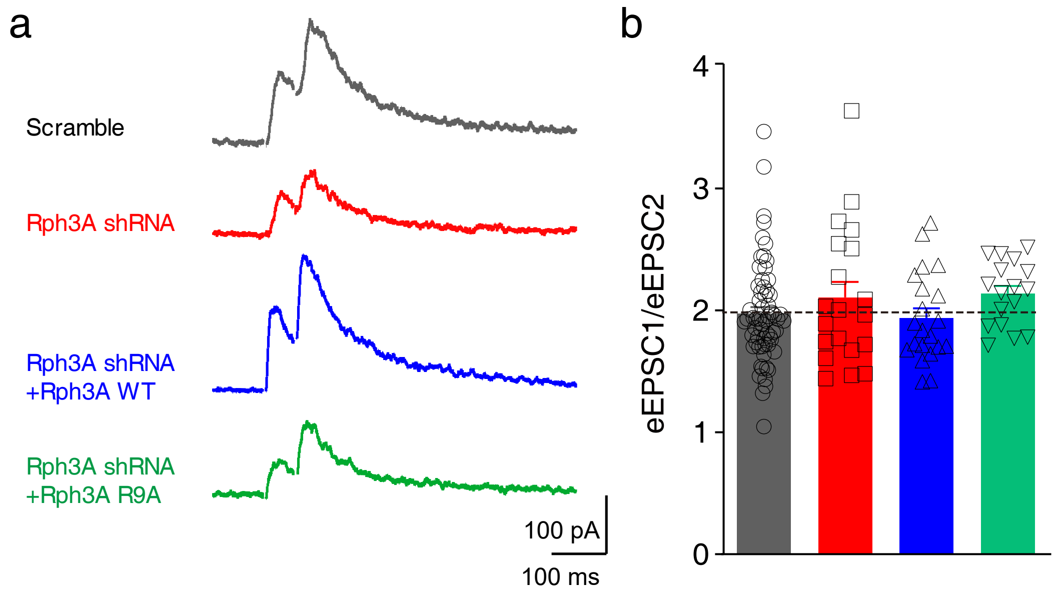

Supplementary Fig. 10: Disruption of the phase separation capacity of Rph3A at postsynaptic sites did not influence presynaptic neurotransmitter release at presynaptic sites.

a Representative traces and **b** quantification of the paired-pulse ratio in Rph3A-knockdown and Rph3A-reexpressing neurons. The data are displayed as the mean ± SEM (Scramble: n = 63 neurons; Rph3A shRNA: 20 neurons; Rph3A shRNA-Rph3A WT: 21 neurons; Rph3A shRNA-Rph3A R9A: 17 neurons, p = 0.31, one-way ANOVA). Source data of b are provided in the Source Data file.

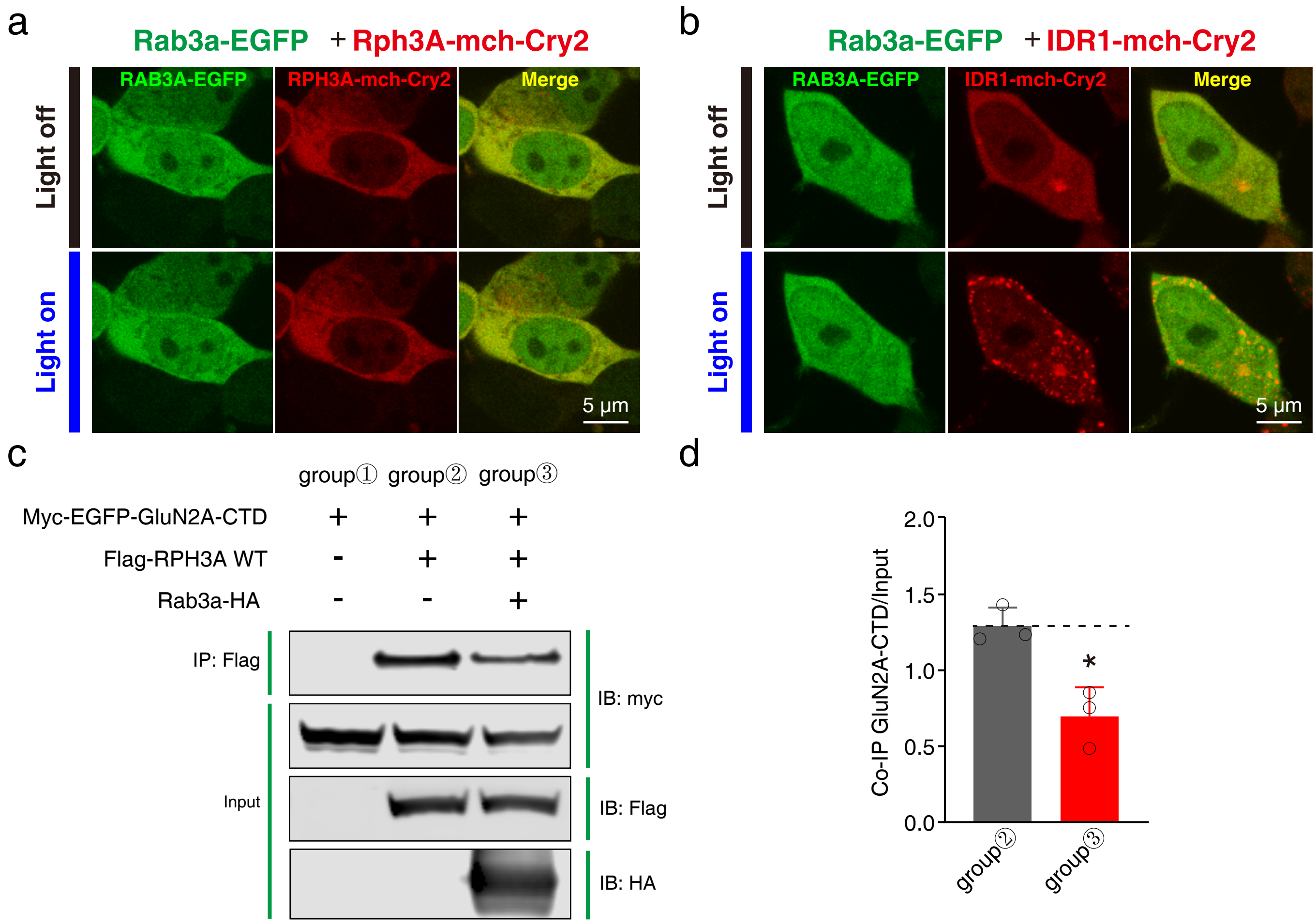

Supplementary Fig. 11: Rab3 abolished the phase separation of Rph3A and impaired the interaction between Rph3A and GluN2A.

a Coexpression of Rab3a abolished droplet formation by Rph3A-mch-Cry2 in the optoDroplet assay. **b** IDR1 of Rph3A, which lacks the Rab3a-binding domain, formed droplets under Rab3a coexpression in the optoDroplet assay. **c, d** Representative images and quantification of the results of the Co-IP of Rph3A with GluN2A CTD in the presence or absence of Rab3a. The data are displayed as the mean ± SEM (n = 3 independent experiments for each group, *$p < 0.01$, two-tailed unpaired t test). Source data, p value and full scan blot image of c and d are provided in the Source Data file.

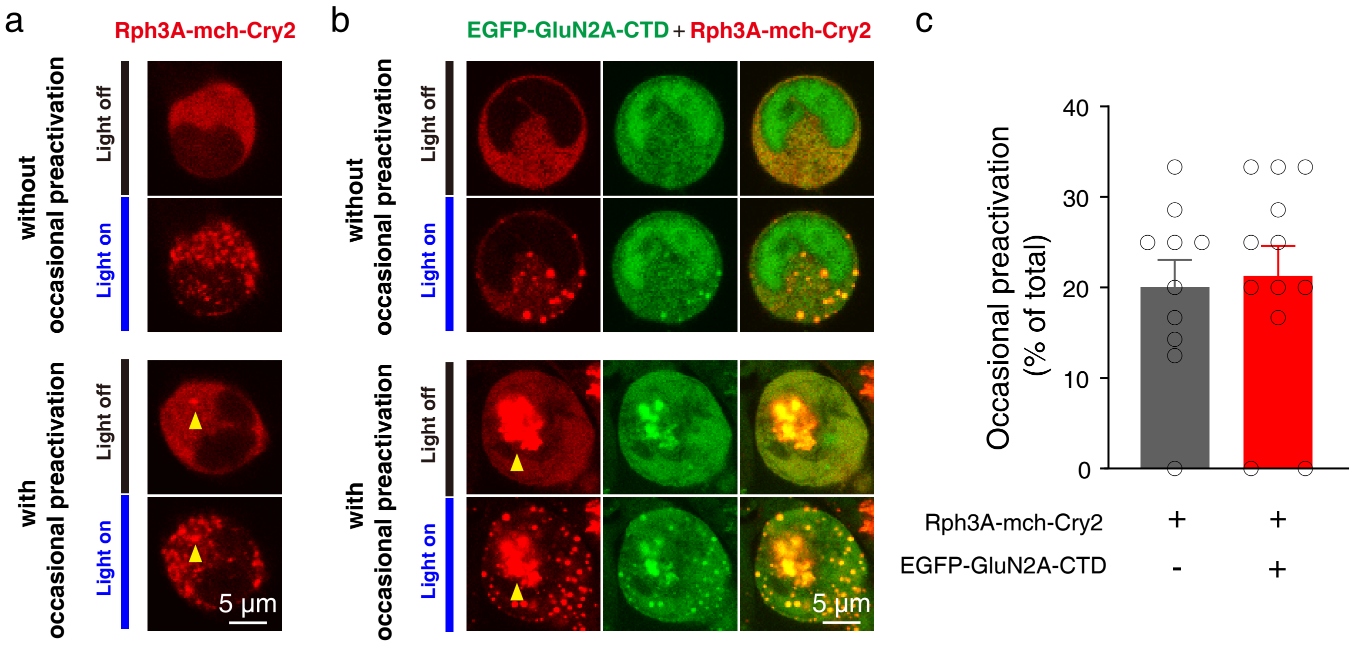

Supplementary Fig. 12: The optoDroplet construct of Rph3A shows occasional preactivation in HEK293 cells.

a, b Representative images showing the occasional preactivation of Rph3A-mch-Cry2 in HEK293 cells. **c** Quantification of the percentage of cells showing occasional preactivation. The data are displayed as the mean ± SEM (n = 10 images for each group, p = 0.79, two-tailed unpaired t test). Source data of c are provided in the Source Data file.

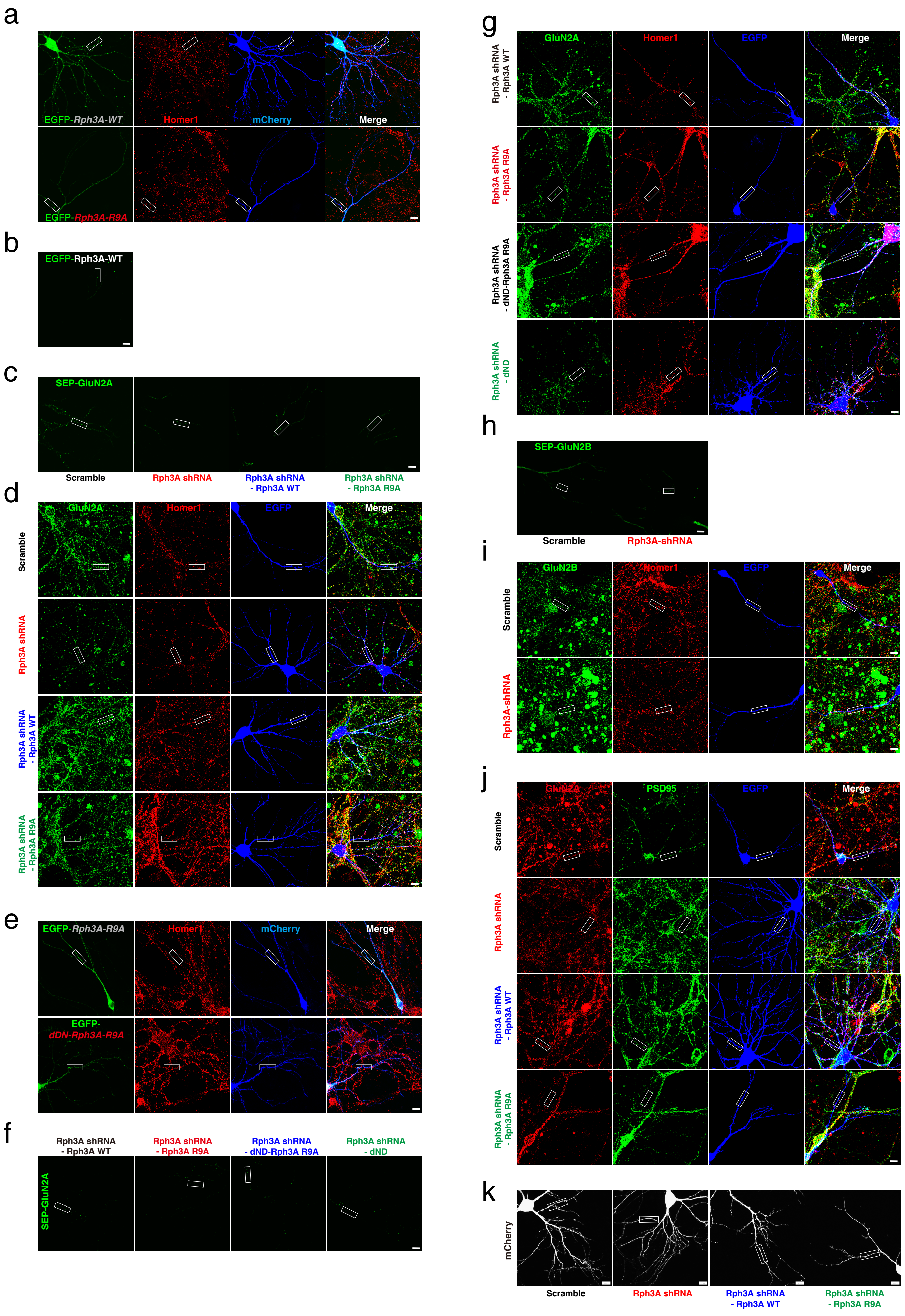

a
EGFP-Rph3A-WT
Homer1
mCherry
Merge
EGFP-Rph3A-R9A
b
EGFP-Rph3A-WT
c
SEP-GluN2A
Scramble
Rph3A shRNA
Rph3A shRNA - Rph3A WT
Rph3A shRNA - Rph3A R9A
d
GluN2A
Homer1
EGFP
Merge
Scramble
Rph3A shRNA
Rph3A shRNA - Rph3A WT
Rph3A shRNA - Rph3A R9A
e
EGFP-Rph3A-R9A
Homer1
mCherry
Merge
EGFP-dDN-Rph3A-R9A
f
Rph3A shRNA - Rph3A WT
Rph3A shRNA - Rph3A R9A
Rph3A shRNA - dND-Rph3A R9A
Rph3A shRNA - dND
SEP-GluN2A
g
GluN2A
Homer1
EGFP
Merge
Rph3A shRNA - Rph3A WT
Rph3A shRNA - Rph3A R9A
Rph3A shRNA - dND-Rph3A R9A
Rph3A shRNA - dND
h
SEP-GluN2B
Scramble
Rph3A-shRNA
i
GluN2B
Homer1
EGFP
Merge
Scramble
Rph3A-shRNA
j
GluN2A
PSD95
EGFP
Merge
Scramble
Rph3A shRNA
Rph3A shRNA - Rph3A WT
Rph3A shRNA - Rph3A R9A
k
mCherry
Scramble
Rph3A shRNA
Rph3A shRNA - Rph3A WT
Rph3A shRNA - Rph3A R9A

Supplementary Fig. 13: The full images of the neuronal segments used as the representative images.

a-d Corresponding full images and selected neuronal segments presented in Fig. 6a, 6b, 6c and 6g. **e-g** Corresponding full images and selected neuronal segments presented in Fig. 7c, 7d, and 7h. **h, i** Corresponding full images and selected neuronal segments presented in Supplementary Fig. 6a and 6e. **j** Corresponding full images and selected neuronal segments presented in Supplementary Fig. 8a. **k** Corresponding full images and selected neuronal segments presented in Supplementary Fig. 9a.
