# Supplementary material for: Rabphilin-3A undergoes phase separation to regulate GluN2A mobility and surface clustering
Source: Nat Commun. 2023 Jan 24;14:379. doi: 10.1038/s41467-023-36046-6 (PMC9873702; doi:10.1038/s41467-023-36046-6)
Supplement: Supplementary file 3 — Reporting Summary [file 41467_2023_36046_MOESM3_ESM.pdf]

nature portfolio

Corresponding author(s): Chen Zhang

Last updated by author(s): Jan 2, 2023

Reporting Summary

Nature Portfolio wishes to improve the reproducibility of the work that we publish. This form provides structure for consistency and transparency in reporting. For further information on Nature Portfolio policies, see our Editorial Policies and the Editorial Policy Checklist.

Statistics

For all statistical analyses, confirm that the following items are present in the figure legend, table legend, main text, or Methods section.

n/a	Confirmed	
☐	☒	The exact sample size (*n*) for each experimental group/condition, given as a discrete number and unit of measurement
☐	☒	A statement on whether measurements were taken from distinct samples or whether the same sample was measured repeatedly
☐	☒	The statistical test(s) used AND whether they are one- or two-sided<br>*Only common tests should be described solely by name; describe more complex techniques in the Methods section.*
☒	☐	A description of all covariates tested
☒	☐	A description of any assumptions or corrections, such as tests of normality and adjustment for multiple comparisons
☐	☒	A full description of the statistical parameters including central tendency (e.g. means) or other basic estimates (e.g. regression coefficient) AND variation (e.g. standard deviation) or associated estimates of uncertainty (e.g. confidence intervals)
☐	☒	For null hypothesis testing, the test statistic (e.g. *F*, *t*, *r*) with confidence intervals, effect sizes, degrees of freedom and *P* value noted<br>*Give P values as exact values whenever suitable.*
☒	☐	For Bayesian analysis, information on the choice of priors and Markov chain Monte Carlo settings
☒	☐	For hierarchical and complex designs, identification of the appropriate level for tests and full reporting of outcomes
☒	☐	Estimates of effect sizes (e.g. Cohen's *d*, Pearson's *r*), indicating how they were calculated

Our web collection on statistics for biologists contains articles on many of the points above.

Software and code

Policy information about availability of computer code

Data collection	Olympus FV3000 viewer was used to collect the images. Clampfit 10.2 (Molecular Devices) was used to collect electrophysiological data.
Data analysis	The Olympus cellSens Standard software (V2.2) was used to analyze the data in FRAP assay. The ImageJ (1.53k) was used in puncta counting, co-localization analysis and quantification of the fluorescence of images. Graphpad Prism (Version 8.0.2 (263)) was used to perform statistics and prepare graphs. DNAMAN (Version 9) was used to align the DNA and protein sequences. Igor 4.0 (Wave Metrics) was used to analyze the electrophysiological data.

For manuscripts utilizing custom algorithms or software that are central to the research but not yet described in published literature, software must be made available to editors and reviewers. We strongly encourage code deposition in a community repository (e.g. GitHub). See the Nature Portfolio guidelines for submitting code & software for further information.

Data

Policy information about availability of data

All manuscripts must include a data availability statement. This statement should provide the following information, where applicable:

- Accession codes, unique identifiers, or web links for publicly available datasets
- A description of any restrictions on data availability
- For clinical datasets or third party data, please ensure that the statement adheres to our policy

The source data generated in this study are provided as a Source Data file with this paper.

Human research participants

Policy information about studies involving human research participants and Sex and Gender in Research.

Reporting on sex and gender	There is no human research participants in this work.
Population characteristics	See above
Recruitment	See above
Ethics oversight	See above

Note that full information on the approval of the study protocol must also be provided in the manuscript.

Field-specific reporting

Please select the one below that is the best fit for your research. If you are not sure, read the appropriate sections before making your selection.

☒ Life sciences ☐ Behavioural & social sciences ☐ Ecological, evolutionary & environmental sciences

For a reference copy of the document with all sections, see nature.com/documents/nr-reporting-summary-flat.pdf

Life sciences study design

All studies must disclose on these points even when the disclosure is negative.

Sample size	No statistical methods were used to predetermine sample size. Sample size of more than 5 in 3 individual experiments was chosen based on those generally employed in the field, which are generally sufficient for statistical test. The precise sample sizes are mentioned in the manuscript.
Data exclusions	No data were excluded from the analyses.
Replication	All the data were collected by 3 individual experiments. All attempts at replication were successful.
Randomization	The experiments were performed based on different proteins and different treatments (gene manipulation) of HEK293 cells or neurons. For data analysis, the HEK293 cells, neurons, droplets are chosen randomly.
Blinding	The investigators responsible for images collection and data analysis were blinded to group allocations.

Reporting for specific materials, systems and methods

We require information from authors about some types of materials, experimental systems and methods used in many studies. Here, indicate whether each material, system or method listed is relevant to your study. If you are not sure if a list item applies to your research, read the appropriate section before selecting a response.

Materials & experimental systems

n/a	Involved in the study	
☐	☒	Antibodies
☐	☒	Eukaryotic cell lines
☒	☐	Palaeontology and archaeology
☐	☒	Animals and other organisms
☒	☐	Clinical data
☒	☐	Dual use research of concern

Methods

n/a	Involved in the study	
☒	☐	ChIP-seq
☒	☐	Flow cytometry
☒	☐	MRI-based neuroimaging

Antibodies

Antibodies used	Anti-NMDAR2A (GluN2A) (extracellular) Antibody (Alomone); catalog number: AGC-002; RRID: AB_2040025.<br>Anti-NMDAR2B (GluN2B) (extracellular) Antibody (Alomone); catalog number: AGC-003; RRID: AB_2040028.<br>Homer 1 antibody (Synaptic Systems); catalog number: 160 011; Clone: 2G8<br>PSD95 antibody (Neuromab);catalog number: 75-028; Clone: K28/74<br>DYKDDDDK-Tag(3B9) mAb (Abmart);catalog number: M20008L;Clone: 3B9

	Myc-Tag (19C2) mAb (Abmart);catalog number: M20002H; Clone: 19C2<br>HA-Tag(26D11) mAb (Abmart);catalog number: M20003L; Clone: 26D11<br>Dylight 649, Goat Anti-Mouse IgG (Abbkine); catalog number: A23610<br>Goat anti-Rabbit IgG (H+L) Highly Cross-Adsorbed Secondary Antibody, Alexa Fluor™ Plus 555(Invitrogen); catalog number: A32732
Validation	Anti-NMDAR2A (GluN2A) (extracellular) Antibody has been validated by Alomone by applicating in Western blot analysis of rat brain lysates, live cell imaging in live intact rat C6 glioma cells and immunohistochemistry in perfusion-fixed frozen mouse brain sections. It was also validated in plenty of publications. (https://www.alomone.com/p/anti-nmda-receptor-2a-glun2a-extracellular/AGC-002)<br>Anti-NMDAR2B (GluN2B) (extracellular) Antibody has been validated by Alomone by applicating in Western blot analysis of rat brain lysates, live cell imaging in hippocampal primary neurons and immunohistochemistry in hippocampal CA1 frozen slice. It was also validated in plenty of publications. (https://www.alomone.com/p/anti-nmda-receptor-2b-glun2b-extracellular/AGC-003).<br>Homer 1 antibody (Synaptic Systems) has been validated by Synaptic Systems by applicating in Western blot analysis of membrane fraction of rat brain, immunostaining of rat hippocampus neurons. It was also validated in plenty of publications. (https://sysy.com/product/160011)<br>PSD95 antibody (Neuromab) has been validated by western blot knockout validation.It was also validated in plenty of publications. (https://www.labome.com/product/Neuromab/75-028.html)<br>DYKDDDDK-Tag(3B9) mAb (Abmart) has been validated by Abmart by applicating in western blot and immunostaining of HEK293T cells overexpressed DYKDDDDK tagged protein.<br>Myc-Tag (19C2) mAb (Abmart) has been validated by Abmart by applicating in western blot and immunostaining of HEK293T cells overexpressed myc tagged protein.<br>HA-Tag(26D11) mAb (Abmart) has been validated by Abmart by applicating in western blot and immunostaining of HEK293T cells overexpressed HA tagged protein.

Eukaryotic cell lines

Policy information about cell lines and Sex and Gender in Research

Cell line source(s)	HEK293, ATCC, CRL-1573<br>Primary cultured hippocampal neurons from C57BL/6J mice (0 day after birth).
Authentication	HEK293 cells were authenticated by STR profiling by the supplier. Primary cultured hippocampal neurons were not authenticated.
Mycoplasma contamination	Cell lines were not tested for mycoplasma contamination but no appearance of contamination was observed.
Commonly misidentified lines<br>(See ICLAC register)	There were no commonly misidentified cell lines.

Animals and other research organisms

Policy information about studies involving animals; ARRIVE guidelines recommended for reporting animal research, and Sex and Gender in Research

Laboratory animals	Wild-type C57BL/6J (0 day after birth) mice was used for primary hippocampal neurons culture.
Wild animals	No wild animals is used in this work.
Reporting on sex	This finding do not apply to only one sex.
Field-collected samples	No field-collected samples is used in this work.
Ethics oversight	All animal studies were conducted according to the Guide for the Care and Use of Laboratory Animals (8th edition) and approved by the Animal Experiments and Experimental Animal Welfare Committee of Capital Medical University (Approval ID: AEEI-2019-013).

Note that full information on the approval of the study protocol must also be provided in the manuscript.
